# Nitrate Transporter Gene Expression and Kinetics of Nitrate Uptake by *Populus* × *canadensis* ‘Neva’ in Relation to Arbuscular Mycorrhizal Fungi and Nitrogen Availability

**DOI:** 10.3389/fmicb.2020.00176

**Published:** 2020-02-28

**Authors:** Fei Wu, Fengru Fang, Na Wu, Li Li, Ming Tang

**Affiliations:** ^1^State Key Laboratory of Conservation and Utilization of Subtropical Agro-bioresources, Guangdong Laboratory of Lingnan Modern Agriculture, Guangdong Key Laboratory for Innovative Development and Utilization of Forest Plant Germplasm, College of Forestry and Landscape Architecture, South China Agricultural University, Guangzhou, China; ^2^College of Forestry, Northwest A&F University, Yangling, China; ^3^Key Laboratory of State Forestry and Grassland Administration on Forest Ecosystem Protection and Restoration of Poyang Lake Watershed, College of Forestry, Jiangxi Agricultural University, Nanchang, China; ^4^School of Life Science, Shanxi Datong University, Datong, China

**Keywords:** arbuscular mycorrhiza, poplar, nitrate transporter gene, nitrate uptake kinetics, nitrogen fertilization

## Abstract

Plants and other organisms in the ecosystem compete for the limited nitrogen (N) in the soil. Formation of a symbiotic relationship with arbuscular mycorrhizal fungi (AMF) may influence plant competitiveness for N. However, the effects of AMF on plant nitrate (NO_3_^–^) uptake capacity remain unknown. In this study, a pot experiment was conducted to investigate the effects of N application and *Rhizophagus irregularis* inoculation on the root absorbing area, uptake kinetics of NO_3_^–^, and the expression of NO_3_^–^ transporter (*NRT*) genes in *Populus* × *canadensis* ‘Neva’. The results showed that *R*. *irregularis* colonized more than 70% of the roots of the poplar and increased root active absorbing area/total absorbing area. The uptake kinetics of NO_3_^–^ by poplar fitted the Michaelis–Menten equation. Mycorrhizal plants had a higher maximum uptake rate (*V*_max_) value than non-mycorrhizal plants, indicating that *R*. *irregularis* enhanced the NO_3_^–^ uptake capacity of poplar. The expression of *NRTs* in roots, namely, *NRT1;2*, *NRT2;4B*, *NRT2;4C*, *NRT3;1A*, *NRT3;1B*, and *NRT3;1C*, was decreased by *R*. *irregularis* under conditions of 0 and 1 mM NH_4_NO_3_. This study demonstrated that the improved NO_3_^–^ uptake capacity by *R*. *irregularis* was not achieved by up-regulating the expression of *NRTs* in roots. The mycorrhizal pathway might repress root direct pathway in the NO_3_^–^ uptake by mycorrhizal plants.

## Introduction

Nitrogen (N) is an essential component of nucleic acids, proteins, and many other critical biomolecules of living organisms (Castro-Rodríguez et al., 2017). Limited N in terrestrial ecosystems generally causes strong competition between different players, such as plants and/or soil microorganisms (Geisseler et al., 2010). Microorganisms are considered to be more competitive than plants in regard to N uptake due to their unique properties, such as faster growth rates and larger surface-area-to-volume ratios (Kuzyakov and Xu, 2013). In order to acquire enough N for growth, plants have evolved several N uptake strategies, including changing their root physiological characteristics and gene expression levels in N uptake systems, and establishing symbiosis with microorganisms (Kraiser et al., 2011).

Several different forms of N can be available to plant roots, such as ammonium (NH_4_^+^), nitrate (NO_3_^–^), and amino acids (Fan et al., 2017). Nitrate is usually the primary form of inorganic N absorbed by plants in aerobic soils (Xu et al., 2012). The NO_3_^–^ uptake rate is mainly determined by NO_3_^–^ uptake kinetics, which can be modeled by the Michaelis–Menten equation, which denotes the absorption rate of NO_3_^–^ as a function of external NO_3_^–^ concentration (McMurtrie and Näsholm, 2017). Two parameters of Michaelis–Menten kinetics, the maximum uptake rate (*V*_max_) and the Michaelis constant (*K*_m_), can describe N absorption characteristics (York et al., 2016). The *V*_max_ value is the maximum influx rate of NO_3_^–^, whereas the *K*_m_ value is the external NO_3_^–^ concentration when the absorption rate is half of *V*_max_ and presents the affinity of transporters (York et al., 2016).

Uptake of NO_3_^–^ from soil is mediated by low- (LATS) and high-affinity transport systems (HATS) (Fan et al., 2017). The molecular basis of LATS and HATS mainly includes two families of NO_3_^–^ transporters (NRT): NRT1/PTR (renamed NPF by Léran et al., 2014) family and NRT2 family (Castro-Rodríguez et al., 2017). The NPF family members are responsible for the LATS, except for NPF6;3/NRT1;1, which has a dual low and high affinity for NO_3_^–^ (von Wittgenstein et al., 2014). Among the 53 known NPF family members in *Arabidopsis*, only AtNPF6;2/AtNRT1;1 and AtNPF4;6/AtNRT1;2 are involved in root NO_3_^–^ influx (Kant, 2018). The NRT2 family has seven members and is responsible for the HATS (von Wittgenstein et al., 2014). The coding genes are induced by a low concentration of NO_3_^–^ and repressed by a high concentration of NO_3_^–^. AtNRT2;1, AtNRT2;2, AtNRT2;4 and AtNRT2;5 have been found playing a role in root uptake of NO_3_^–^ from soil (Kant, 2018). Of these, AtNRT2;1, in interaction with a NO_3_^–^ assimilation-related protein (NAR2/NRT3), accounts for approximately 75% of the high-affinity NO_3_^–^ absorption (Filleur et al., 2001).

Arbuscular mycorrhizal fungi (AMF), belonging to the monophyletic phylum Glomeromycota, are the most widespread root symbionts and can colonize at least 80% of terrestrial plants (Smith and Read, 2008). AMF acquire up to 20% of their host photosynthates and provide water and mineral nutrients in return (Bago et al., 2000; Kiers et al., 2011). Many ^15^N-labeling experiments confirmed the ability of AMF to transport N from soil to plants (Govindarajulu et al., 2005; Jin et al., 2005). Plants can acquire large amounts of N through the mycorrhizal pathway (Smith and Smith, 2011). The effects of AMF on the expression of *NRT* genes have been observed in several plant species, for example, in grapevine (Balestrini et al., 2017), *Solanum lycopersicum* (Ruzicka et al., 2012), and *Lotus japonicus* (Guether et al., 2009). The arbuscular mycorrhizal (AM)-induced alterations in *NRTs* expression suggest that AMF may affect plant uptake capacity for NO_3_^–^. However, the impacts of AMF on plant NO_3_^–^ uptake kinetics are poorly understood.

Poplars have great ecological and economic values and can form symbiotic relationships with AMF (Wu et al., 2017). With the publication of the genome of *Populus trichocarpa* (Tuskan et al., 2006), poplar has become a model specie for woody plant research (Jansson and Douglas, 2007). In the genome database of *P*. *trichocarpa*, 68 NPF/NRT1 and 11 NRT2/NRT3 have been putatively identified (Willmann et al., 2014). The genes *NRT1;1*, *NRT1;2*, *NRT2;4B*, and *NRT2;4C* of poplar were the closest homologs to *AtNRT1;1, AtNRT1;2*, *AtNRT2;4*, and *AtNRT2;1* of *Arabidopsis*, respectively; and *NRT3;1A*, *NRT3;1B*, and *NRT3;1C* of poplar were the closest homologs to *AtNRT3;1* of *Arabidopsis* (Li et al., 2012; Luo et al., 2013). These genes may play important roles in NO_3_^–^ uptake by poplar because their expression levels are markedly affected by changes in available N (Li et al., 2012; Gan et al., 2015). The objectives of the current research were to (i) evaluate the effects of AMF on NO_3_^–^ uptake kinetics of poplar and (ii) investigate the influence of AMF on *NRTs* expression under different N levels. The results may provide new insights into the parts AMF play in plant N uptake capacity.

## Materials and Methods

### Plant Material and Fungal Inoculum

Cuttings (6 cm long) of *Populus* × *canadensis* ‘Neva’ were obtained from a plant nursery in Yangling, Shaanxi Province, China. The cuttings were surface sterilized with 75% ethanol and rinsed with sterile water. After surface sterilization, each cutting was planted in a plastic pot (10 × 8 cm) containing 450 g of growth substrate.

*Rhizophagus irregularis* (B109) was commercially provided by the Institute of Plant Nutrition and Resources, Beijing Academy of Agriculture and Forestry Sciences, China. The fungal inoculum contained colonized root fragments, hyphae, spores (approximately 50 spores g^–1^), and cultured sands.

### Growth Substrates

The growth substrate consisted of topsoil (0–20 cm) collected from a poplar field in Yangling, Shaanxi Province, China. Before being put into the pots, the growth substrate was sieved (2 mm) and autoclaved (0.11 MPa, 121°C, 2 h). The soil contained soil organic matter, 18.97 mg kg^–1^; available N, 38.62 mg kg^–1^; available phosphorus, 12.31 mg kg^–1^; and available potassium, 140.26 mg kg^–1^. The pH value was 7.7 (1:5, soil:water).

### Experimental Design and Growth Conditions

The experiment was conducted in a greenhouse using a randomized complete block design with two AM status (inoculated with *R*. *irregularis* or not inoculated with *R*. *irregularis*) and five N levels (0, 1, 5, 10, or 15 mM NH_4_NO_3_), with 30 replicates per treatment. For AM fungal inoculation, each pot received 20 g of inoculum, whereas the non-mycorrhizal treatment received 20 g of autoclaved inoculum and the filtrate (<20 μm) of the inoculum to provide a typical microbial population free of AM propagules (Wu et al., 2017). The cuttings were grown at a temperature of 25–30°C with 12 h of light per day. Ninety days after planting, N application started. NH_4_NO_3_ solution was applied to the corresponding treatments every other day. The N treatment lasted for 28 days. The pots were kept at field capacity during the experiment.

### AM Colonization

The fresh roots (<2-mm diameter) were cut into 1- to 2-cm fragments and stained with trypan blue according to the method from Phillips and Hayman (1970). The proportion of root length colonized by AMF was estimated under a light microscope (×200 magnification; Olympus, Tokyo, Japan) using the gridline intersection method (McGonigle et al., 1990). The arbuscular, vesicular, hyphal, and total colonization values were expressed as a percentage of the length of root fragments containing arbuscules, vesicles, hyphae, and all AMF structures, respectively.

### Root Absorbing Area

The root absorbing area was determined by a methylene blue colorimetric method (Liu et al., 2014). The roots were sequentially immersed for 90 s in three beakers containing a known volume of 0.2 mM methylene blue solution. Then, the concentration and volume of the remaining methylene blue solution in each beaker were determined by a spectrophotometer (UV mini 1240; Shimadzu, Kyoto, Japan) at 660 nm. Methylene blue was used as the standard. The equations are as follows:

Total absorbing area (m^2^) = [(*C* - *C*_1_) × *V*_1_] + [(*C* - *C*_2_) × *V*_2_] × 1.1

Active absorbing area (m^2^) = [(*C* - *C*_3_) × *V*_3_] × 1.1

where *C* (mg mL^–1^) was the initial concentration of the methylene blue solution; *C*_1_, *C*_2_, and *C*_3_ (mg mL^–1^) were the concentrations of the methylene blue solution after three root immersions; *V*_1_, *V*_2_, and *V*_3_ (mL) were the volumes of the methylene blue solution after three root immersions.

### Determination of NO_3_^–^ Uptake Kinetics

Plant NO_3_^–^ uptake was measured using the solution depletion method (Gobert and Plassard, 2002) with some modifications. The roots of the intact plants were carefully washed and transferred to a 0.2 mM CaSO_4_ solution for pretreatment for 48 h. After pretreatment, the plants were transferred to a Ca(NO_3_)_2_ solution (containing 0, 0.01, 0.05, 0.1, 0.2, 0.5, or 1 mM NO_3_^–^) for 6 h. The NO_3_^–^ concentration in the Ca(NO_3_)_2_ solution was determined by an AA_3_ continuous flow analytical system (AA_3_ autoanalyzer; Bran + Luebbe GmbH, Norderstedt, Germany) (Hu et al., 2016). The uptake rates were calculated as follows:

Uptake rates = (*C*_1_ - *C*_2_)/(*t* × *m*)

where *C*_1_ is the initial concentration of NO_3_^–^ (μmol mL^–1^), *C*_2_ is the final concentration of NO_3_^–^ (μmol mL^–1^), *t* is the absorption time (h), and *m* is the root dry weight (g). The kinetic parameters, *V*_max_ and *K*_m_, were calculated using a non-linear curve-fitting model (Michaelis–Menten kinetics) using the Enzyme Kinetics Module of SigmaPlot 13.0 (SPSS Inc., Chicago, IL, United States).

### Quantitative Real-Time PCR Analysis

At harvest, the root and leaf samples were immediately snap-frozen in liquid N and stored at −80°C. RNA was extracted using the cetyl trimethyl ammonium bromide method (Shao et al., 2014). A DNAse treatment was performed to remove residual DNA by using RNase-free DNase I (Invitrogen, Carlsbad, CA, United States). The quantity and quality of RNA were analyzed by measuring the A260/A280 ratio with a spectrophotometer (NanoDrop 2000c; Thermo Scientific, Pittsburgh, PA, United States). The first-strand cDNA was synthesized with 2 μg of total RNA, using a RevertAid first-strand cDNA Synthesis Kit (Thermo Fisher Scientific, Waltham, MA, United States). The gene-specific primers used are listed in Table 1. Quantitative real-time polymerase chain reaction (PCR) was performed on a Bio-Rad CFX96 real-time PCR instrument (Bio-Rad, Hercules, CA, United States). The reactions were performed in 20-μL volumes containing 10 μL of SYBR Premix Ex Taq II (TaKaRa, Dalian, China), 0.8 μL of gene-specific primers, 1.0 μL of cDNA template and 8.2 μL of sterile distilled water. The PCR amplification conditions were 94°C for 10 s and then 40 cycles at 58°C for 20 s and 72°C for 30 s. The ubiquitin gene (*UBQ*) was selected as an internal standard (Couturier et al., 2007). Four biological replicates were utilized for each assay. The relative RNA levels of each gene were determined using the 2^–ΔΔC^_T_ method (Livak and Schmittgen, 2001).

**TABLE 1:** Primers used for quantitative real-time PCR analysis.

Gene name	Primer-forward (5′–3′)	Primer-reverse (5′–3′)
*NRT1;1*^ a^	CTAAACCAAGGGAGGCTCCATGAT	CCCAACACAAAAGTAGGCGAAAAG
*NRT1;2*^ a^	TCTTTGGTAGCAACTTGAACAA	TCTCTCTCTCTCTCGTCTCCCT
*NRT2;4B*^ a^	AATAGAGGAAGGGAATGGCTG	TGAGGTTGTCCCGAATGATAG
*NRT2;4C*^ a^	CAGTCCCGACAGATACAAC	CTTCCCACTACAACGATTTC
*NRT3;1A*^ a^	CCCACTGCCACGTACTTCATA	CAACATGGCACCCACTACTTGT
*NRT3;1B*^ a^	TCATAGCCTCTTCTTCTACCTTTCC	CCACCTTTCAATACTTGTCCG
*NRT3;1C*^ a^	AGAGGTCTCAGTGAAGCGAACAAG	CGCAAATACAAACGCAATTATCAT
*UBQ*^ b^	GCACCTCTGGCAGACTACAA	TAACAGCCGCTCCAAACAGT

*Primer sequences were from ^*a*^Li et al. (2012) and ^*b*^Couturier et al. (2007).*

### Statistical Analyses

Experimental data were statistically analyzed using the statistical software package SPSS 13.0 (SPSS Inc., Chicago, IL, United States). The data were tested for normality and homogeneity of variances. A two-way analysis of variance (ANOVA) was used to analyze the effects of N treatment and AMF treatment and their interactions on the measured parameters, at a significance level of *P* = 0.05. The means were compared by Tukey’s multiple comparison test (*P* = 0.05). Figures were drawn using SigmaPlot 10.0 (Systat Software, San Jose, CA, United States).

## Results

### AM Colonization

**FIGURE 1:**
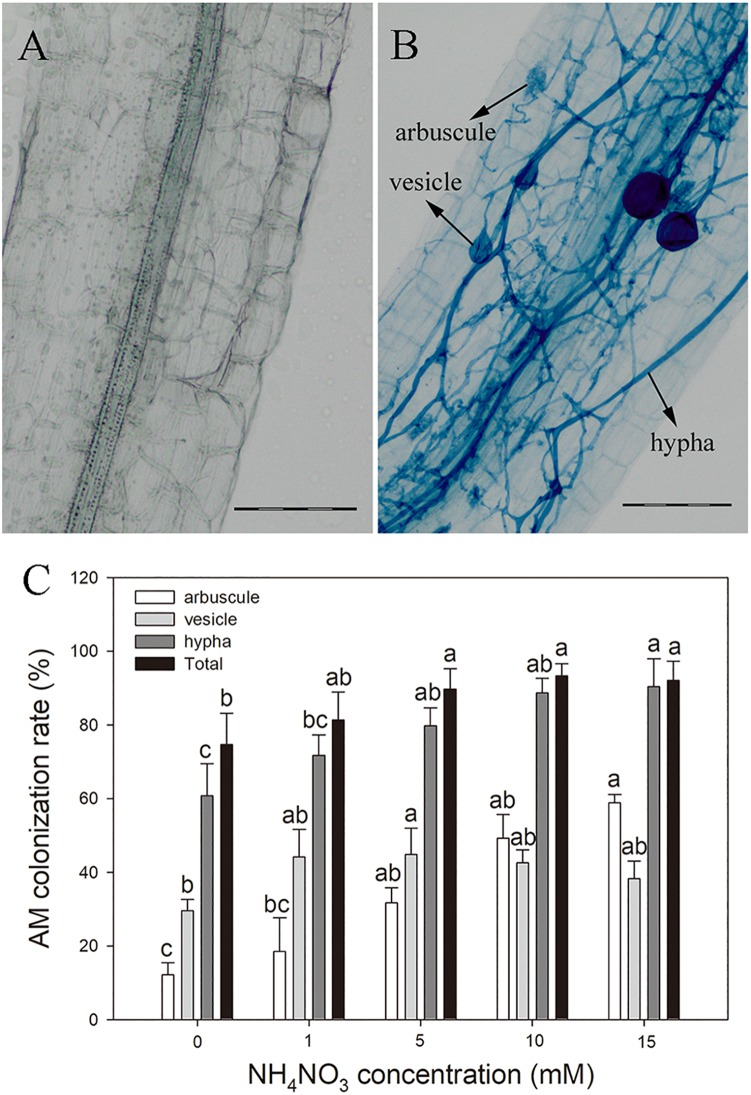
Arbuscular mycorrhizal fungi structures in non-inoculated roots **(A)** and *Rhizophagus irregularis*–inoculated roots of poplar **(B)** and the AM colonization rates under different nitrogen levels **(C)**. Values are presented as means ± SD (*n* = 3). Means followed by the same letter do not differ significantly at *P* < 0.05 by Tukey’s test. Scale bar = 100 μm.

AM colonization structures were observed in more than 70% of the poplar roots after inoculation with *R*. *irregularis*, whereas no AM structure was observed in NM plants (Figure 1). The hyphal colonization was prominent in mycorrhizal poplar roots (Figure 1C). With increasing N level, the arbuscular, hyphal, and total colonization rates increased substantially. The highest arbuscular and hyphal colonization rates (58.85 and 90.42%, respectively) were observed in plants treated with 15 mM NH_4_NO_3_.

### Root Absorbing Area

According to the two-way ANOVA, the total absorbing area was notably affected by N treatment and the interaction of N treatment and AMF treatment (Table 2). The active absorbing area was affected by N treatment, AMF treatment, and their interaction. The ratio of active absorbing area to total absorbing area was affected by AMF treatment. Nitrogen fertilization increased the total absorbing area and active absorbing area in both NM and AM plants (Table 3). The active absorbing area/total absorbing area of AM plants was higher than that of NM plants under each N level (3.47, 2.51, 2.73, 3.65, and 2.21% under 0, 1, 5, 10, and 15 mM NH_4_NO_3_, respectively).

**TABLE 2:** Effects of nitrogen treatment (N), AMF treatment (AMF), and N × AMF on the physiological parameters and expression levels of nitrate transporter genes of poplar.

Parameters	N		AMF		N × AMF	
	*F*	*P*	*F*	*P*	*F*	*P*
Total absorbing area	2496.90	0.00**	1.37	0.26^ns^	3.93	0.02*
Active absorbing area	568.02	0.00**	597.54	0.00**	357.72	0.00**
Active absorbing area/total absorbing area	2.03	0.13^ns^	39.96	0.00**	0.36	0.84^ns^
*V*_max_	23.75	0.00**	44.74	0.00**	0.37	0.83^ns^
*K*_m_	8.63	0.00**	1.56	0.23^ns^	0.56	0.70^ns^
*NRT1;1* (leaf)	62.10	0.00**	31.12	0.00**	6.84	0.00**
*NRT1;2* (leaf)	5.39	0.00**	20.94	0.00**	2.23	0.09^ns^
*NRT2;4B* (leaf)	20.67	0.00**	3.71	0.06	23.63	0.00**
*NRT2;4C* (leaf)	5.63	0.00**	0.30	0.59	1.57	0.21
*NRT3;1A* (leaf)	5.40	0.00**	53.94	0.00**	15.88	0.00**
*NRT3;1B* (leaf)	103.03	0.00**	164.53	0.00**	80.57	0.00**
*NRT3;1C* (leaf)	258.62	0.00**	373.58	0.00**	187.58	0.00**
*NRT1;1* (root)	31.05	0.00**	23.77	0.00**	13.52	0.00**
*NRT1;2* (root)	45.76	0.00**	54.13	0.00**	26.78	0.00**
*NRT2;4B* (root)	123.35	0.00**	125.58	0.00**	55.95	0.00**
*NRT2;4C* (root)	8.16	0.00**	18.81	0.00**	1.45	0.24^ns^
*NRT3;1A* (root)	14.78	0.00**	38.09	0.00**	3.97	0.01*
*NRT3;1B* (root)	223.50	0.00**	115.53	0.00**	67.93	0.00**
*NRT3;1C* (root)	145.86	0.00**	17.31	0.00**	20.89	0.00**

^ *^P < 0.05; ^**^P < 0.01; ^ns^non-significant.

**TABLE 3:** Effects of N fertilization and AMF inoculation on root total absorbing area, active absorbing area, and active absorbing area/total absorbing area of poplar.

NH_4_NO_3_ (mM)	AMF	Total absorbing area (m^ 2^)	Active absorbing area (m^ 2^)	Active absorbing area/total absorbing area (%)
0	NM	12.54 ± 0.15^ c^	6.26 ± 0.14^ d^	49.91 ± 0.76^ bc^
	AM	12.86 ± 0.37^ c^	6.64 ± 0.09^ a^	51.64 ± 0.91^ a^
1	NM	19.13 ± 0.48^ b^	9.52 ± 0.19^ c^	49.76 ± 0.46^ b c^
	AM	19.76 ± 0.25^ b^	10.08 ± 0.09^ b^	51.01 ± 1.00^ ab^
5	NM	25.50 ± 0.16^ a^	12.63 ± 0.19^ a^	49.54 ± 0.43^ bc^
	AM	25.76 ± 0.27^ a^	13.11 ± 0.17^ a^	50.89 ± 0.40^ ab^
10	NM	25.78 ± 0.21^ a^	12.65 ± 0.20^ a^	49.09 ± 0.38^ c^
	AM	25.78 ± 0.16^ a^	13.12 ± 0.09^ a^	50.88 ± 0.69^ ab^
15	NM	26.23 ± 0.18^ a^	12.93 ± 0.06^ a^	49.30 ± 0.17^ c^
	AM	25.63 ± 0.40^ a^	12.92 ± 0.09^ a^	50.39 ± 0.56^ a bc^

*Values are presented as means ± SD (*n* = 3); within columns, values followed by the same lowercase letter do not differ significantly at *P* < 0.05 by Tukey’s test. NM, non-mycorrhizal; AM, arbuscular mycorrhizal.*

### NO_3_^–^ Uptake Kinetics

**FIGURE 2:**
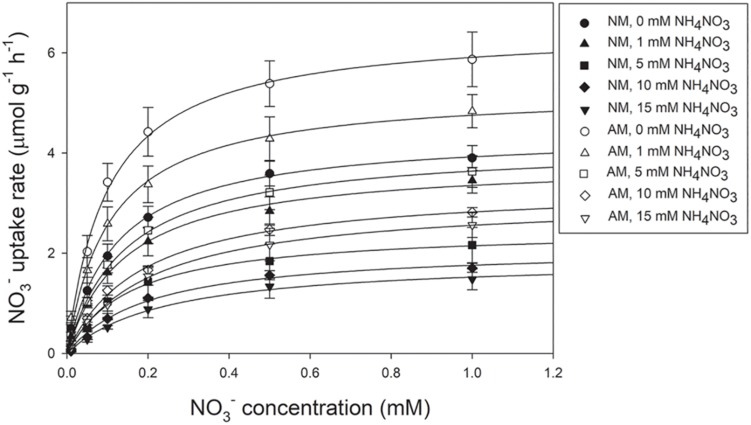
The NO_3_^ –^ uptake rates of poplar as a function of NO_3_^ –^ solution concentration. Values are presented as means ± SD (*n* = 3). NM, non-mycorrhizal; AM, arbuscular mycorrhizal.

The NO_3_^–^ uptake rates of both AM and NM plants increased with increasing external NO_3_^–^ concentrations (Figure 2). The NO_3_^–^ uptake rates were fitted using a non-linear regression of the Michaelis–Menten equation (*V* = *V*_max_/(*K*_m_ + [NO_3_^–^]). According to the two-way ANOVA results, *V*_max_ values were affected by N treatment and AMF treatment, whereas *K*_m_ values were affected by N treatment (Table 2). The N × AMF interaction had no effect on the *V*_max_ and *K*_m_ values. Nitrogen decreased *V*_max_ values but increased *K*_m_ values (Table 4). *V*_max_ values were higher in AM plants than in NM plants under each N level (46.83, 37.24, 67.07, 86.11, and 64.02% under 0, 1, 5, 10, and 15 mM NH_4_NO_3_, respectively). *K*_m_ values were not significantly different between AM and NM plants.

**TABLE 4:** The kinetic parameters of NO_3_^ –^ absorption in the roots of poplar.

N	AMF	*V*_max_	*K*_m_	*R*
treatment	treatment	(μmol g^ – 1^ h^ – 1^)	(mM)	
0	NM	4.42 ± 0.38^ bc^	0.13 ± 0.05^ abc^	0.92
	AM	6.49 ± 1.09^ a^	0.10 ± 0.02^ c^	0.91
1	NM	3.84 ± 0.52^ cd^	0.15 ± 0.03^ a bc^	0.92
	AM	5.27 ± 0.57^ a b^	0.11 ± 0.03^ bc^	0.90
5	NM	2.49 ± 0.72^ d e^	0.16 ± 0.02^ a bc^	0.79
	AM	4.16 ± 0.67^ bcd^	0.18 ± 0.07^ abc^	0.93
10	NM	1.80 ± 0.83^ e^	0.21 ± 0.06^ ab c^	0.97
	AM	3.35 ± 0.21^ cde^	0.19 ± 0.03^ abc^	0.98
15	NM	1.89 ± 0.56^ e^	0.24 ± 0.02^ a^	0.86
	AM	3.10 ± 0.53^ c de^	0.21 ± 0.03^ ab^	0.90

*Values are presented as means ± SD (*n* = 3); within columns, values followed by the same lowercase letter do not differ significantly at *P* < 0.05 by Tukey’s test. NM, non-mycorrhizal; AM, arbuscular mycorrhizal.*

### *NRTs* Expression in Leaves and Roots

**FIGURE 3:**
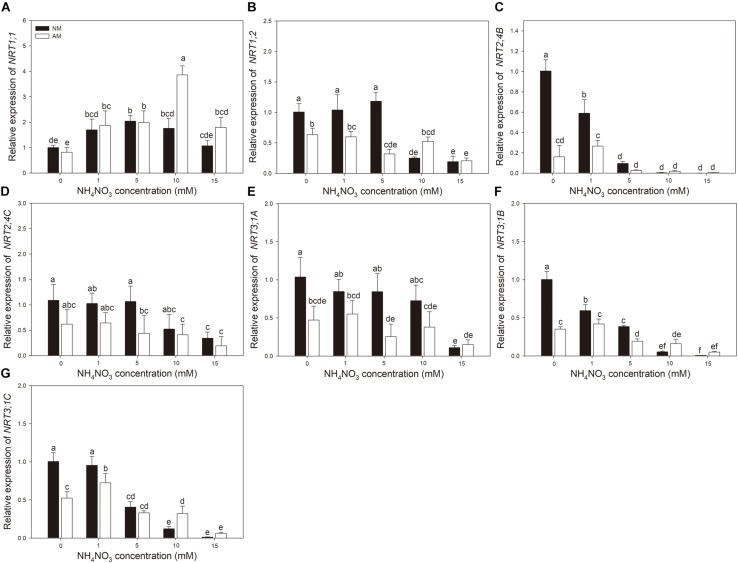
**(A–G)** *NRT1;1*, *NRT1;2*, *NRT2;4B*, *NRT2;4C*, *NRT3;1A*, *NRT3;1B*, and *NRT3;1C* expression levels in roots of mycorrhizal and non-mycorrhizal poplar in response to increasing nitrogen concentrations. Values are presented as means ± SD (*n* = 4). Means followed by the same letter do not differ significantly at *P* < 0.05 by Tukey’s test. NM, non-mycorrhizal; AM, arbuscular mycorrhizal.

**FIGURE 4:**
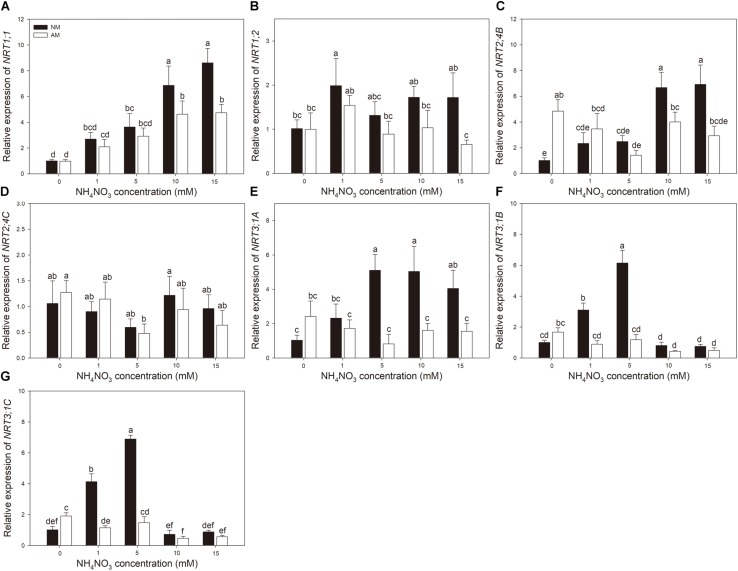
**(A–G)** *NRT1;1*, *NRT1;2*,*NRT2;4B*, *NRT2;4C*, *NRT3;1A*, *NRT3;1B*, and *NRT3;1C* expression levels in leaves of mycorrhizal and non-mycorrhizal poplar in response to increasing nitrogen concentrations. Values are presented as means ± SD (*n* = 4). Means followed by the same letter do not differ significantly at *P* < 0.05 by Tukey’s test. NM, non-mycorrhizal; AM, arbuscular mycorrhizal.

Nitrogen treatment significantly affected the expression of all the tested N transporter genes (Table 2). AMF treatment significantly affected the expression of *NRT1;1*, *NRT1;2*, *NRT3;1A, NRT3;1B*, and *NRT3;1C* in leaves and *NRT1;1*, *NRT1;2*, *NRT2;4B, NRT2;4C, NRT3;1A, NRT3;1B*, and *NRT 3;1C* in roots. The interaction of N treatment and AMF treatment significantly affected the expression of *NRT1;1, NRT2;4B*, *NRT3;1A, NRT3;1B*, and *NRT 3;1C* in leaves and *NRT1;1*, *NRT1;2*, *NRT2;4B, NRT3;1A, NRT3;1B*, and *NRT3;1C* in roots. In roots, the expression of *NRT2;4B*, *NRT2;4C*, *NRT3;1A*, *NRT3;1B*, and *NRT3;1C* in NM plants decreased with increasing N application (Figure 3). In leaves, the expression of *NRT1;1* and *NRT2;4B* increased with increasing N application (Figure 4). AMF reduced the expression of *NRT1;2*, *NRT2;4B*, *NRT2;4C*, *NRT3;1A*, *NRT3;1B*, and *NRT3;1C* in roots (Figure 3) and up-regulated the expression of *NRT2;4B* in leaves under 0 and 1 mM NH_4_NO_3_ (Figure 4). Under 10 and 15 mM NH_4_NO_3_, AMF up-regulated the expression of *NRT1;1* in roots and reduced the expression of *NRT1;1*, *NRT1;2*, and *NRT2;4B* in leaves. Correlation analysis showed that the expression levels of *NRT3;1A*, *NRT3;1B*, and *NRT3;1C* were positively correlated with the expression levels of *NRT1;2*, *NRT2;4B*, and *NRT2;4C* (Table 5).

**TABLE 5:** Correlation coefficients between the expression levels of the nitrate transporter genes in roots of poplar.

Index	*NRT1;1*	*NRT1;2*	*NRT2;4B*	*NRT2;4C*
*NRT3;1A*	−0.088	0.666*	0.664*	0.798*
*NRT3;1B*	−0.262	0.777*	0.943*	0.709*
*NRT3;1C*	−0.173	0.744*	0.859*	0.655*

Pearson correlation coefficients *r* were determined across all nitrogen treatments and inoculation treatments (*n* = 40). *P < 0.017 (Bonferroni correction).

## Discussion

The formation of symbiosis is the starting point of AMF to improve plant nutrient uptake and stress resistance (Zhang et al., 2017). In this study, the structures of arbuscules, vesicles, and internal hyphae were clearly observed inside the roots of mycorrhizal poplar (Figure 1). More than 70% of the roots of the *P*. × *canadensis* ‘Neva’ cuttings were colonized by *R*. *irregularis*, which accords with previous findings that *P*. × *canadensis* ‘Neva’ was capable of establishing AM symbiosis (Liu et al., 2014). Nitrogen application influences AMF colonization rate, but inconsistent results have been reported (Calabrese et al., 2017; Wheeler et al., 2017). Using the same intersection method as this study, Wheeler et al. (2017) found a negative effect of chronic N application on the AMF colonization rate of *Acer rubrum*, whereas Calabrese et al. (2017) observed the opposite result in *P*. *trichocarpa*. The higher photosynthetic capacity in N-applied plants may provide more photosynthates for AMF and may promote AMF colonization (Wu et al., 2017).

The establishment of AM in roots can trigger the changes in root activities and gene expression patterns of root systems, for example, in grapevine (Balestrini et al., 2017; Torres et al., 2018), *Robinia pseudoacacia* (Huang et al., 2019), *L*. *japonicus* (Guether et al., 2009), and *Lycopersicon esculentum* (Hildebrandt et al., 2002). The results of this study showed that the active absorbing area/total absorbing area ratios of AM plants were higher than those in NM plants, which was in keeping with observations by Liu et al. (2014) and Huang et al. (2019). An increase in root activities suggests a stronger element uptake capacity (Yang et al., 2008). Uptake of NO_3_^–^ by plants relies on NO_3_^–^ transport systems (Garnett et al., 2010). In this study, the absorption of NO_3_^–^ by *P*. × *canadensis* ‘Neva’ followed Michaelis–Menten kinetics, as with other plant species, for example, *Solanum tuberosum* (Sharifi and Zebarth, 2006), *Fagus sylvatica* (Kreuzwieser et al., 2000), *Populus tremuloides* Michx., and *Pinus contorta* Dougl. ex Loud. var. *latifolia* Engelm. (Min et al., 2000). Nitrogen application decreased the *V*_max_ value and increased the *K*_m_ value, which was consistent with previous studies showing that HATS was under feedback repression by N levels, and was down-regulated by N supply and up-regulated by N limitation (Bassirirad, 2000; Rothstein et al., 2000). Inoculation with *R*. *irregularis* increased the *V*_max_ value of poplar in absorption of NO_3_^–^, irrespective of N treatment. So far, the effects of AMF on the kinetic analysis of NO_3_^–^ uptake have never been reported. In ectomycorrhizal research, inoculation with *Rhizopogon roseolus* increased the *V*_max_ of *Pinus pinaster* for NO_3_^–^ absorption, indicating that ectomycorrhiza conferred plants with higher ability to utilize NO_3_^–^ (Gobert and Plassard, 2002). Kuzyakov and Xu (2013) found a higher *V*_max_ and smaller *K*_m_ for NO_3_^–^ in microorganisms than in plants, indicating that microorganisms have an advantage in the absorption of NO_3_^–^, especially at low concentrations. In general, microorganisms absorb the majority of N immediately after N is mineralized from soil organic matter (Kuzyakov and Xu, 2013). The higher NO_3_^–^ uptake rates in AM plants than in NM plants were observed regardless of the external NO_3_^–^ concentration, suggesting that AMF increased the competitive ability of the host to use fluctuating concentrations of NO_3_^–^ in the soil solution (Gobert and Plassard, 2002; Kuzyakov and Xu, 2013).

Regulation of NO_3_^–^ transporters is an essential factor for elucidating NO_3_^–^ uptake and assimilation. Nitrate transport systems have been well identified in *Arabidopsis* (Kant, 2018). AtNPF6;2/AtNRT1;1 was found to be a dual-affinity NO_3_^–^ transporter, participating in low- and high-affinity NO_3_^–^ uptake and function as a NO_3_^–^ sensor (Liu et al., 1999; Ho et al., 2009). AtNPF4;6/AtNRT1;2 was found to be a low-affinity NO_3_^–^ transporter, and *AtNPF4;6*/*AtNRT1;2* has been observed being constitutively expressed in root NO_3_^–^ uptake (Huang et al., 1999). AtNRT2;1 is the main NO_3_^–^ inducible member of the HATS, whereas NRT2;2 plays a minor role in NO_3_^–^ absorption (Li et al., 2007). AtNRT2;5 has a similar role to AtNRT2;4, which plays a main role in NO_3_^–^ uptake under N-starvation condition (Kiba et al., 2012; Lezhneva et al., 2014). Moreover, the interaction of NRT2 members with NAR2;1 (NRT3;1) is necessary to function in the NO_3_^–^ transport capacity of NRT2 (Okamoto et al., 2006; Tsay et al., 2007). The results of this study showed that the expression levels of *NRT2;4B*, *NRT2;4C*, *NRT3;1A*, *NRT3;1B*, and *NRT3;1C* in the roots of poplar were negatively correlated with external NO_3_^–^ concentrations, supporting the suggestion that NRT2 family members are high-affinity transporters (Li et al., 2012). The positive correlations between the expression levels of *NRT3;1A*, *NRT3;1B*, or *NRT3;1C* and the expression levels of *NRT1;2*, *NRT2;4B*, or *NRT2;4C* indicated that they might combine to function in NO_3_^–^ absorption (Tsay et al., 2007). In leaves, the expression of *NRT1;1* and *NRT2;4B* were up-regulated by N application, which may be due to a higher amount of N transferred to leaves with increasing N application. Under N-limiting condition, the transcript levels of *NRTs* in roots increased, and the expression of *NRTs* in leaves decreased, which may be a possible mechanism for poplar adaptation to an N-deficient environment (Luo et al., 2013).

Previous studies reported that AMF can influence NO_3_^–^ uptake of plants by regulating the transcript levels of *NRTs* (Chen et al., 2018; Hildebrandt et al., 2002). With transcriptomics techniques, Balestrini et al. (2017) demonstrated that *Funneliformis mosseae* up-regulated the expression of two putative NO_3_^–^ transporters in the roots of grapevine. In *L. japonicus*, four *NRTs* showed fourfold to 18-fold up-regulation by *Gigaspora margarita* colonization (Guether et al., 2009). Tian et al. (2017) found both up- and down-regulated expression of *NRTs* in the roots of *Triticum aestivum* by *R*. *irregularis* colonization. The differential responses of *NRTs* to AM colonization may be related to the different functions of *NRTs*. Some *NRTs* induced by AMF were expressed in arbuscule-containing cortical cells and may be involved in symbiosis development or N transfer through the periarbuscular membrane (Gaude et al., 2012). Hildebrandt et al. (2002) found that *LeNRT2;3* was expressed in inner cortical cells of *L. esculentum*, in which arbuscules and vesicles were mainly distributed. The expression of *LeNRT2;3* was higher in AMF-colonized roots than in non-colonized controls, which probably mediated the positive effects of AMF on NO_3_^–^ uptake from soil and the distribution of NO_3_^–^ to the host (Hildebrandt et al., 2002). In this research, the absorption capacity of poplar for NO_3_^–^ was improved by *R*. *irregularis*, but no AM-specific NRT was detected. The expression levels of *NRT1;2*, *NRT2;4B*, *NRT2;4C*, *NRT3;1A*, *NRT3;1B*, and *NRT3;1C* were lower in *R*. *irregularis*–colonized poplar roots compared with non-colonized roots at 0 and 1 mM NH_4_NO_3_. The mycorrhizal plants have two N absorption pathways, the root direct pathway and the mycorrhizal pathway (Smith and Smith, 2011). Plants that form AM may use the mycorrhizal pathway to absorb NO_3_^–^ and down-regulate the transcript levels of *NRTs* involved in the root direct pathway (Tian et al., 2017). Ruzicka et al. (2012) demonstrated that, under low inorganic N concentrations in the soil, the N uptake of mycorrhizal tomatoes shifted toward mycorrhizal-mediated N uptake over the root direct pathway. *NRT1;2*, *NRT2;4B*, *NRT2;4C*, *NRT3;1A*, *NRT3;1B*, and *NRT3;1C* may be involved in the root direct pathway and repressed in the presence of the mycorrhizal pathway (Ruzicka et al., 2012).

The diverse N sources and N conversion reactions lead to complex N pools in the field, of which organic N is the largest N pool (Kuypers et al., 2018). Most organisms prefer bioavailable N forms, such as NH_4_^+^ and NO_3_^–^, for which production is mainly controlled by microbial reactions (Kuypers et al., 2018). Nitrate is the main inorganic N form in aerobic soils (Xu et al., 2012). Compared with microorganisms, plants have shown a lower *V*_max_ and higher *K*_m_ for NO_3_^–^, which indicated a disadvantage in competing for NO_3_^–^ (Kuzyakov and Xu, 2013). Previous studies demonstrated that AMF could change the microbial community that degrades organic matter and promote plant uptake of inorganic N produced during the degradation of organic matter (Hodge and Fitter, 2010; Nuccio et al., 2013). This study showed that AMF improved the ratio of root active absorbing area/total absorbing area and increased the *V*_max_ value of the Michaelis–Menten equation for NO_3_^–^ uptake of poplar, resulting in a promotion in NO_3_^–^ uptake capacity. The key genes in NO_3_^–^ uptake of poplar were repressed by AMF under 0 and 1 mM NH_4_NO_3_, indicating that the mycorrhizal pathway may have an impact on the root direct pathway for NO_3_^–^ uptake. These results implied that the formation of AM symbiosis may be an efficient strategy for plants to compete with microorganisms for the finite available N in the soil. However, the NO_3_^–^ uptake kinetics of *R*. *irregularis* remains unclear (Kuzyakov and Xu, 2013). Further studies are needed to investigate the NO_3_^–^ uptake characteristics of AMF, in order to elucidate the mechanism of AMF in plant N uptake.

## Data Availability Statement

All datasets generated for this study are included in the manuscript.

## Author Contributions

FW, FF, and MT designed the experiments. FW, FF, NW, and LL performed the experiments. FW and FF analyzed the data. FW and MT wrote the manuscript.

## Conflict of Interest

The authors declare that the research was conducted in the absence of any commercial or financial relationships that could be construed as a potential conflict of interest.
